# Prenatal Screening, Reproductive Choice, and Public Health

**DOI:** 10.1111/bioe.12121

**Published:** 2014-12-17

**Authors:** Stephen Wilkinson

**Keywords:** prenatal, screening, public health, autonomy, reproduction, eugenics, consent

## Abstract

One widely held view of prenatal screening (PNS) is that its foremost aim is, or should be, to enable reproductive choice; this is the Pure Choice view. The article critiques this position by comparing it with an alternative: Public Health Pluralism. It is argued that there are good reasons to prefer the latter, including the following. (1) Public Health Pluralism does not, as is often supposed, render PNS more vulnerable to eugenics-objections. (2) The Pure Choice view, if followed through to its logical conclusions, may have unpalatable implications, such as extending choice well beyond health screening. (3) Any sensible version of Public Health Pluralism will be capable of taking on board the moral seriousness of abortion and will advocate, where practicable, alternative means of reducing the prevalence of disease and disability. (4) Public Health Pluralism is at least as well-equipped as the Pure Choice model to deal with autonomy and consent issues.

## 1. Introduction

Promoting informed choice is commonly recognized as the chief purpose and benefit of prenatal screening, its very presence being viewed as a key way in which the process can be distanced from eugenics.^1^

As Clare Williams and her colleagues note (above), one widely held view of prenatal screening (PNS) is that its aim is, or should be, to enable reproductive choice. This is the Pure Choice model. According to this view, PNS is importantly different from most other screening programmes, where the aim is not choice *per se* but rather to improve public health (for example, by allowing the detection, prevention, and treatment of disease) or to reduce healthcare costs.^2^

Why might one think that the proper goal of PNS is choice? Two main answers are offered. First, it is suggested that there is something ethically problematic about governments attempting to achieve health goals by encouraging or facilitating abortion. Antina de Jong and her colleagues, for example, state that:

Enabling meaningful reproductive choice with regard to parenting or avoiding a child with a serious disorder or disability is (or should be) the very aim of offering testing for fetal abnormalities. This is in order to ensure that abortion decisions remain personal and are not turned into instruments of societal goals, such as prevention and cost-reduction by bringing down the number of people requiring life-long and costly care.^3^

Second, it is argued that PNS programmes are less vulnerable to objections couched in terms of disability rights and eugenics if their purpose is to enable choice, rather than to reduce the prevalence of disability:

If informed decisions rather than abortion rates are taken as a measure of success, the disability rights critique is less convincing.^4^

This article proceeds as follows. Section 2 describes two possible views about PNS: the Pure Choice view just mentioned, and an alternative termed Public Health Pluralism. It then proceeds to offer some reasons for preferring the latter. Section 3 looks at a possible objection to Public Health Pluralism: de Jong's claim that we must ‘ensure that abortion decisions remain personal and are not turned into instruments of societal goals’.^5^ Finally, Section 4 looks at consent issues: specifically the claim that, since it is (allegedly) difficult for many women to adequately understand (and validly consent to) prenatal tests, we should be wary about making such tests part of public health policy.

## 2. Pure Choice and Public Health Pluralism

This section does three things. First, it describes the differences between the Pure Choice view and Public Health Pluralism. Second, it examines the supposition that the Pure Choice view is relatively immune to charges of disability discrimination and eugenics. Finally, it raises a potential problem for the Pure Choice view: the suggestion that it may have unpalatable implications.

### 2.1 Public Health Pluralism

Public Health Pluralism comprises two basic tenets:

(a) that the best rationale for a systematic state-supported PNS programme includes several different elements (hence ‘pluralism’); and
(b) that amongst the most important of those elements are public health goals (hence ‘public health’).

The Pure Choice view denies both (a) and (b). It denies (a) because it says that there is only a single aim only: providing choice. It denies (b) because choice is not a public health aim. Proponents of the Pure Choice view could argue that promoting choice *is* a public health aim (in which case they would deny only (a)) but this is implausible, chiefly because adding choice seems to be a good which is independent of health and, while there are situations in which adding choice may promote health (adding contraceptive options perhaps), there are equally situations in which it can diminish health (e.g. if access to alcohol and other recreational drugs were to become easier). The ensuing discussion then assumes that, although providing choice may well be a good thing and a legitimate aim for national health services, providing choice is not in itself a public health aim, because it is not necessarily linked to positive health outcomes.^6^

That, then, is a schematic definition of Public Health Pluralism. Within this view, or family of views, there are then many possible variants depending on (amongst other things) what list of goals is attached to PNS and what view of public health is taken. For now, I am going merely to suggest that the following is a plausible list of goals (in no particular order):

(i) improving population health, by reducing the prevalence of disability and disease in the new-born population;
(ii) improving maternal and fetal health;
(iii) reducing future health and social welfare costs and/or increasing the cost-effectiveness of future health and social welfare spending (either through (i) and (ii), or as an independent goal)
(iv) respecting autonomy, requiring valid consent (where practicable), and providing choice (where appropriate).^7^

(ii) is relatively uncontroversial. (i) and (iii) are more controversial for reasons explored later. (iv) is noteworthy because it allows the Public Health Pluralist to agree with many proponents of the Pure Choice view that autonomy, choice, and consent are important goods. Thus, on the account sketched here, both positions can attach positive value to autonomy and choice. The difference between them is that the Public Health Pluralist view *also* sees things other than choice as amongst the most important aims of a state-supported PNS system.

### 2.2 Disability Discrimination and Eugenics

One of the main concerns about using PNS and abortion to ‘select out’ disability is that it is allegedly eugenic and sends out an unacceptably negative message to and about people with disabilities. As Tom Shakespeare puts it:

… it may be claimed that prenatal diagnosis discriminates against disabled children and adults, because it sends the message that it would have been better if they, too, had not been born. The argument is often called ‘the expressivist objection’, because it suggests that genetic diagnosis and selective abortion ‘expresses’ discriminatory or negative views towards disabled people.^8^

Public health and health-economic approaches to PNS policy are thought by some to be especially vulnerable to this kind of criticism because they explicitly aim to reduce the prevalence of disability in the new-born population.^9^ The Pure Choice model, on the other hand, is often thought to be less vulnerable. As de Jong et al. put it:

If informed decisions rather than abortion rates are taken as a measure of success, the disability rights critique is less convincing.^10^

But is this really the case? As far as eugenics is concerned, many people have made the point that (depending of course on how it is defined) eugenics need not be state eugenics and need not be authoritarian. King, for example, states that:

In the conventional definition, the key aspect of eugenics is coercion of people's reproductive choices, for social ends, which may include ‘improving the quality of the population’, ‘preventing suffering of future generations’ or reducing financial costs to the state. […] However, examination of the history of eugenics reveals that coercion is certainly not one of its defining characteristics. From its very beginnings, many eugenicists, including the founder of the eugenics movement, Francis Galton, were opposed to coercion.^11^

King has a point. While one *could* define eugenics in a way that makes state coercion an essential element, this would be out of keeping with much present and historical usage of the term. So it is at least arguable that ‘liberal eugenics’ is as properly eugenic, so to speak, as ‘authoritarian eugenics’ although (as I have argued elsewhere) it is not clear that there is necessarily much wrong with liberal eugenics, provided that the means used to pursue it are not themselves unethical.^12^

Similar considerations apply to the beliefs about disability on which PNS is based and the message that it sends out. That is, it can be plausibly argued that there is not much difference, as far as negative attitudes to disability are concerned, between a screening programme which aims to reduce the prevalence of disability and one that merely aims to provide choice, if it is known that most people, when given a choice, choose to avoid disability. Down's syndrome is a good example here, as it has been reported that around 90% of Down's syndrome diagnoses in the UK result in termination.^13^

Consider, for example, a parallel with fetal sex selection and imagine two scenarios. In the first, the state aims to reduce the prevalence of girls and encourages this by offering sex testing early in pregnancy. In the second, the state merely aims to give families choice and so offers sex testing early in pregnancy, but knows full well that, because the population is sexist, 90% of people will choose to abort girls. As far as the message sent out about the value of women is concerned, it might plausibly be argued that there is not much difference between these two scenarios.

‘Disability rights’ critics may say that PNS for disability is just like the sex selection case in this respect. Given that (for example) we know that 90% of prospective mothers will choose to end their pregnancies following a diagnosis of Down's syndrome, in practice (they may argue) there is little difference between a system that aims only to promote choice and one which aims directly to reduce the prevalence of Down's, since reduced prevalence of Down's is the known outcome in both cases. It may also be argued that just as providing sex testing would be a way of aiding and abetting sexist parents in their attempts to get rid of female fetuses, screening for disability is similarly a way of aiding and abetting disablist parents in their attempts to avoid having a child with a disability.

I have argued elsewhere that selecting out disability is, in some important respects, different from sex selection.^14^ So I do not accept all of this ‘disability critique’ of PNS. I do however accept the specific point just raised, which is that there is not a fundamental difference – at least as far as the ‘message sent out’ to people with disabilities is concerned – between merely providing choice in a situation where that will predictably lead to the mass ‘selecting out’ of disabled fetuses and having a PNS programme which explicitly aims (through non-coercive and consensual means) to reduce the prevalence of disability in the new-born population.

Against this, it may be argued that there is a morally significant difference between, on the one hand, actively supporting ‘selecting out’ disability and, on the other, merely allowing it to happen. But, even if there is such a distinction, this will make little difference to the overall line of argument here, for the matter under consideration is not the state's *merely allowing* ‘selecting out’ disability. Rather, the concern of this article is systematic state-supported PNS programmes. Thus, an appeal to a doing/allowing or acts/omissions distinction is not going to bite in this case, since the state is ‘active’ in both cases (supporting pure choice or supporting selecting out disability).

Similarly, it may be argued that there is a morally significant distinction between the state's, on the one hand, intentionally reducing the prevalence of disability in the new-born population and its, on the other, bringing about the same result as a merely foreseen but unintended side-effect. Quite how generally significant this kind of distinction is has been the subject of considerable debate in moral philosophy over many years.^15^ But, even leaving aside any general concerns that one might have about ‘double effect’, the distinction will not take us very far in this case.

One reason for this is that we are talking about states and not individual human beings and assigning intentions to intangible entities like states is liable to be especially problematic. How do we work out what a state intends and what it merely foresees? Must we do so by looking inside the minds of presidents, of ministers, of the parliamentarians who voted for particular measures, or even of the electorate? Or is state intention an impersonal emergent property to be divined through a more abstract interpretative process? These are just questions and I do not argue here that it is impossible to ascribe intentions to states. My point rather is that, especially in contested policy areas, this may be very hard to do. And specifically in relation to the area under consideration here, disability rights advocates may well say that even if the *stated aim* of government policy is not to eliminate people with disabilities, this is nonetheless the government's *unstated intention*. Whether this is true will vary from case to case but the general form of the critique is sound: in particular, the suggestion that we cannot just assume that governments' *explicitly stated* goals are their *real* goals.

A second reason why ‘double effect’ may not get us very far is that, if preventing the births of children with disabilities is as morally problematic as many critics suppose, then the state would have an obligation not merely to avoid *intentionally preventing* those births, but also a stronger or wider obligation to ensure that its policies *do not foreseeably cause* people to stop children with disabilities from being born. Consider, for example, a parallel with child abuse. It would not be much of a defence for the government to say that its policies are defensible because, although they foreseeably cause child abuse, the abuse is not what the government intended. The same then could go for detecting and aborting disabled fetuses. If (*and this is a ‘big if’*) that is seriously morally wrong, then stating that it is (‘merely’) a foreseeable effect of policy, rather than something that the government intends, is not going to be a compelling defence.

So when it comes to deciding between a Pure Choice and a Public Health Pluralist Model (one which allows the rationale to be some combination of providing choice, reducing the prevalence of disability, reducing costs, and acting to benefit pregnant women) it seems that the latter is not necessarily any more vulnerable to eugenics-critiques than the former. This is because, firstly, it is at least arguable that ‘liberal eugenics’ is still eugenics and hence ‘pure choice’ can have eugenic effects and can allow prospective parents to enact eugenic preferences. Secondly it is because, in terms of the effects on the disabled population and the ‘message sent out’, there may be little practical difference between a system which aims to reduce the prevalence of disability, and one which aims only to promote choice but with the knowledge that the vast majority of women will use that choice to ‘select against’ disability.

### 2.3 Does Pure Choice have unpalatable implications?

Another challenge for the Pure Choice model is that, if we were to concede that choice is the sole justification for PNS, that raises the question of why the choices offered should be limited to serious health conditions and disabilities. If choice is the fundamental good, why not offer pregnant women as much choice as possible?

One answer to this, one that could come from within the Pure Choice paradigm, is to say that simply maximizing the number of available options is not necessarily the best way of maximizing *meaningful* choice, or of respecting *autonomy* (which is what matters more fundamentally). This suggestion is comparable to what people sometimes say about certain kinds of market failure. For example, in the UK, there is presently a public debate about whether householders are being made to choose between too many different complex energy tariffs for energy. According to a 2009 *Which*? Report:

… baffling bills and an array of complex tariffs make it very difficult for consumers to understand what gas and electricity schemes they are using and reduce their energy consumption and costs. Which? is calling on energy suppliers, the Government and Ofgem [the energy regulator] to take immediate steps to simplify bills and tariffs.^16^

Allegedly the complex array of tariffs makes it less likely that consumers will engage with the market and less likely that they will make good choices. For this reason, some political parties are proposing that consumer choice should be reduced (i.e. there should be fewer tariffs) in order to simplify the market and to make it more likely that householders will make good choices. One might then say something similar about PNS. If women are offered too many choices, too many tests, they may become confused and either make bad choices, or fail to engage with the decision-making process and uncritically accept the views of their doctors or midwives.

What should we make of this argument? Well, it is an argument which in principle could count against a very radical ‘individualized choice’ model of PNS: one in which women are offered a wide and potentially confusing array of tests.^17^ Whether however this argument works in practice depends on how complex and difficult to grasp the various options are, on how the relevant information is presented, and on how well-equipped different women are to comprehend the information. These are all matters about which it would be unwise to generalize in the absence of solid empirical evidence.

Also, returning to the potential problem for the Pure Choice view (the suggestion that, if choice is the fundamental good, why not offer pregnant women as much choice as possible?) it is not clear that this argument about autonomy and complexity is going to save the Pure Choice view from the initial objection, because some of the choices at stake are not very complicated.

Take sex selection again. Whether one's baby will be a boy or a girl is a pretty simple thing to grasp and sex testing is pretty straightforward. So, if someone believes in the Pure Choice view, shouldn't she also believe that women should be provided with fetal sex testing with the option of termination if the fetus is not of the desired sex? Or if fetal testing were possible for eye or hair colour or height, shouldn't pregnant women be offered the opportunity to end those pregnancies too if it looks as if their future child will not have the desired characteristics?

A natural response to these suggestions is to say that abortion is a morally serious business and that the health service is justified in supporting screening and abortion only where the woman has a *very good* reason for ending a pregnancy, such as the detection of serious fetal abnormality, as opposed to a *trivial* reason, such as a preference about sex or cosmetic features. Now this response may or may not be right; whether it is or not is a huge issue and not one that can be tackled here. But, even if it is right, crucially it is not one that is available to proponents of the Pure Choice view. If the sole reason for allowing testing and termination is to provide women with choices (rather than some other reason, like women's interests, public health, or cost-saving) then there is no basis for treating fetal disability differently from fetal sex; for the justification that applies to screening and termination in the case of disability (‘freedom to choose’) applies equally to screening and termination in the case of sex selection. As Ann Furedi puts it:

We either support women's capacity to decide, or we don't. You can't be pro-choice except when you don't like the choice, because that's not pro-choice at all.^18^

This seems highly plausible. If choice is the ultimate value appealed to, it cannot just apply to those choices we approve of. Hence, proponents of the Pure Choice view face a dilemma. Either they must restrict the range of choices that they are willing to support and face charges of inconsistency (only supporting the ‘choices that they like’), *or* they must apply their pro-choice view consistently and support screening and termination for reasons many would regard as trivial and unacceptable.

If this is a problem for the Pure Choice view, does the Public Health Pluralist view fare any better? Yes it does, for the Public Health Pluralist view can appeal to other goals or values to differentiate sex selection (for example) from ‘selecting out’ fetal disease and disability. In particular, as we have seen, a central tenet of Public Health Pluralism is that PNS programmes should aim to reduce the prevalence of disease and disability in the new-born population; this clearly provides a basis on which to say that screening for fetal abnormalities is justified, whereas arguably screening for sex and cosmetic features is not. So, in this respect, the Public Health Pluralism is preferable to Pure Choice.

It is important also to note again that I am talking here about whether or not the state should actively support (by funding and/or providing) particular types of PNS – not merely about whether (for example) sex-selective abortion should be *legally permitted*. This makes the argument just outlined more powerful than it otherwise would be. For if we were just talking about the legal permissibility of sex selection, then proponents of the Pure Choice view could easily ‘bite the bullet’ and say that sex selection should be allowed. This is not a popular view but it is one that could conceivably be defended within a generally liberal ethico-legal framework.^19^ However, if we are talking about state funding and support then consistency requires proponents of the Pure Choice view to go further and to advocate not merely the legal permissibility of sex-selective abortion, but the state funding of this as well: a position that many proponents of Pure Choice will find unappealing.

## 3. Ensuring that Abortion Decisions ‘Remain Personal’

This section addresses a concern about using PNS to achieve public health goals (and hence a possible objection to Public Health Pluralism): de Jong et al.'s claim that we must:

… ensure that abortion decisions remain personal and are not turned into instruments of societal goals, such as prevention and cost-reduction by bringing down the number of people requiring life-long and costly care.^20^

There are two worries here. One (the subject of this section) is that, given the morally controversial and sensitive nature of abortion, the state should not use it as a means of achieving its goals (be they public health or anything else); while a second (considered in the next section) is that using PNS and abortion to achieve public health goals may generate consent problems.

Much of the ethical concern about PNS programmes relates to the fact that they achieve their goals primarily by increasing the frequency of abortion for fetuses with impairments. PNS programmes can improve public health in other ways as well (for example, by taking targeted steps during pregnancy to improve maternal or fetal health, or by enabling parents to prepare themselves to care for a sick or disabled child) but the most significant aspect is selective termination.

Some people are opposed to all (or nearly all) abortion and believe that the state should not even be allowing, let alone encouraging or funding, abortions whether connected to PNS or not. This is not an obviously incoherent position. However, it must be side-lined for the present because such concerns are too general to underpin an argument specifically against PNS (and *a fortiori* against a specific model of PNS). These arguments are not what we need because they count against all abortion, rather than specifically against using PNS and selective termination.

Having side-lined very general anti-abortion arguments, what others remain? One is that, where practicable, the state should use means other than abortion to achieve public health goals. Another related thought is that ‘earlier’ selective reproduction is always to be preferred to ‘later’ (other things being equal). Thus preconception selection (e.g. of gametes or gamete donors) is preferable to preimplantation embryo selection, which in turn is preferable to ‘earlier’ abortion, which in turn is preferable to ‘later’ abortion. This is a widely held view and one that coheres with a plausible gradualist view of embryonic or fetal moral status. As de Jong et al. put it:

… the dominant opinion in most western countries, often also reflected in legislation, is that the moral status of the embryo/fetus progressively increases with its development (the gradualist view).^21^

These points however are perhaps less arguments against PNS and selective termination, and more ethical side-constraints on policy.

Take Down's syndrome again and let us assume (at least for the sake of argument) that reducing the prevalence of Down's in the new-born population is a legitimate public health goal. There are various different (non-mutually exclusive) ways in which this goal might be achieved and we can order these in terms of ‘earlier’ or ‘later’ positions in the reproductive process. Earliest of all are preconception interventions, such as encouraging women not to delay reproduction until their forties, or folic acid supplementation (which is also a possible during-pregnancy intervention).^22^ Next, at the embryonic stage, we have the possibility of IVF with pre-implantation genetic diagnosis, which could be used to screen out affected embryos.^23^ Finally, there is antenatal detection using blood tests and/or ultrasound scans, combined with the possibility of termination and then, within antenatal detection, we may differentiate between earlier and later tests.

The point of the example is that there is no reason why a supporter of PNS for Down's syndrome couldn't *also* support pre-conception measures to avoid Down's, and (where appropriate) PGD to avoid Down's. What is more, she may well prefer these interventions (especially the preconception ones) because they are going to be less traumatic for the woman and bypass ethical concerns about abortion. However, pre-conception and pre-implantation measures are not always available, practicable, or acceptable to the reproductive community. In the case of Down's, for example, persuading women to have children when they are younger is not going to be easy, and it would be wrong to exert undue pressure on the over-forties not to reproduce, while IVF/PGD may not be worthwhile for all but the most ‘high risk’ couples. In these circumstances, it will often be necessary to fall back on PNS as the only effective and feasible strategy for Down's prevention.

So the proper role of the undesirability of abortion in the argument seems to be this. Supporters of PNS can and should concede that, where practicable, means other than PNS and abortion should be used to achieve public health goals. Leading alternatives include informing prospective parents about lifestyle and nutritional changes that reduce their risk of conceiving a disabled fetus, and embryo selection within the context of IVF. But, given that these ‘earlier’ means may not always be available, or may carry with them other disadvantages, there is nonetheless still a role (in practice, a significant role) for PNS.

In addition, other things being equal, ‘earlier’ versions of PNS (i.e. earlier within pregnancy) are to be preferred to ‘later’ ones. So if, as seems reasonably likely, scientists manage to develop a non-invasive prenatal test for Down's that is just as accurate as today's tests and yet can be administered earlier on in pregnancy, then such a development is to be welcomed.^24^ Or at least it is to be welcomed as far as concerns about abortion are concerned; it may carry with it other disadvantages.^25^

So to conclude: these concerns about abortion do not entail that we must never use PNS and selective termination as a means of achieving public health goals. However, any sensible version of Public Health Pluralism (and any ethical PNS programme) will take on board the moral seriousness and sensitivity of abortion and will advocate, where practicable, alternative means of reducing the prevalence of disease and disability; but where there is no practicable alternative, facilitating selective abortion may still be justified. As regards the particular concern about abortion decisions ‘remaining personal’, proponents of both Pure Choice and Public Health Pluralism can agree that this is first and foremost a matter of ensuring that pregnant women's choices and consents are sufficiently voluntary and informed. That is the issue to which I now turn.

## 4. Information, Understanding, and Consent

The foundation of the contemporary provision of prenatal screening and diagnosis is the recognition of patients' individual right[s] to decide whether or not they wish to receive testing and then to make reproductive choices based on test results. At a minimum, informed consent requires that patients have sufficient relevant information and that their decisions are voluntary and uninfluenced by external pressures whether they be medical insurance, societal, or political.^26^

Another set of concerns relates to consent. The general form of the worry is that if governments or national health services seek to reduce the prevalence of disability and disease in future generations by encouraging and providing PNS, this will create cases in which pregnant women's consents (to screening and testing, and/or to termination) are defective or invalid. One version of this argument says that often pregnant women are not sufficiently well-informed to give valid consent. This could be either because the information provided by the healthcare system is inadequate or biased, or because the array of tests and choices on offer to pregnant women is too complex for them to understand adequately. A second version says that sometimes pregnant women's consents are not sufficiently voluntary because of general social pressure; it is argued that unduly negative social attitudes to disability, alongside a lack of social support for those caring for disabled children, combine to dissuade pregnant women from continuing their pregnancies once fetal disability has been detected.

This article focuses just on the first version (the claim that there is a problem with information or understanding) and will say very little about the voluntariness argument. This is because if general societal pressure to avoid having a disabled child is the source of a consent problem, then this is something with implications for all reproductive decisions with a disability dimension, regardless of which particular model of PNS is adopted. In other words, if there is undue general social pressure to avoid becoming the parent of child with a disability, then this will be *at least* as much a problem (as far as consent is concerned) under a Pure Choice model as it is under a Public Health Pluralist model. So there is a sense in which this concern functions at too general a level to engage with the specific interests of this article. Also, the academic debate about consent, coercion, and ‘social pressure’ is large and complex and has been covered extensively elsewhere; so, for reasons of space and focus, it is best not simply to rehash that material here.^27^

Turning then to information and understanding, it is perhaps tempting initially to give this issue fairly short shrift: not because it is not important, but just because there is little reason to think that the information issues faced by pregnant women undergoing antenatal screening are fundamentally different from those faced by patients in any other part of the health service. Thus for pregnant women, as for many other patient groups, there will be cases in which the information provided by healthcare professionals leaves a lot to be desired, because it is biased or insufficient. Benn and Chapman, for example, state that:

Issues have been raised about the completeness, accuracy, and bias in the information physicians currently communicate to mothers who have received a prenatal diagnosis of Down syndrome … Also, while professional societies' guidelines subscribe to the norm of nondirective counselling, some physicians and genetic professionals admit to overemphasizing the negative aspects of Down syndrome or even urging pregnant women to seek a termination.^28^

We should of course be ready to condemn poor consent practice wherever it occurs and to seek improvement and, as Rafi and Chitty note, particularly in the light of developments such as non-invasive prenatal diagnosis, ‘we will need to maintain high standards of counselling to facilitate informed consent taking account of cultural variation’.^29^ But it is hard to see either why this is an inevitable flaw in PNS, or why this carves it out as different from any other area of healthcare delivery. Also it is worth noting that, at least as a matter of official policy, bodies like the UK National Health Service go out of their way to be non-directive about conditions like Down's syndrome.^30^

It is however worth looking at another variant of the information argument. Whereas the concerns considered so far focussed on failings in the health services, this alternative argument suggests instead that some aspects of screening are in practice impossible for many patients to understand, even if their healthcare professionals' consent practice is excellent. One of the main things that supposedly is too hard to grasp is the inevitably probabilistic and uncertain nature of some prenatal tests. Milligan, for instance, cites ‘poor understanding of the probabilistic and statistical language of risk’.^31^ Many screening tests are not capable of showing with near certainly that the fetus does or does not have Condition X. Rather what they show is that there is a 75% of its having Condition X, an 85% chance of Condition Y, and a 50% chance of Condition Z, etc..

Clearly *some* women have a good understanding of probability and risk in pregnancy, not least because female obstetricians and statisticians themselves become pregnant from time to time. So the argument only applies, if it works at all, to that majority of women whom (it is supposed) have a poor understanding of probability and risk. There is then a major empirical question here about just how poor that majority's understanding is. I do not propose to tackle that here except to note that it does seem plausible to suppose that people generally have a reasonably poor grasp of probabilities expressed as percentages and of risk. And, at least for the sake of argument let's allow that this is the case, in general and specifically in relation to prenatal testing. What would follow from that?

One way to go is to have a not very demanding conception of informed consent and to say that, so long as the healthcare professionals have *provided* the relevant information in a reasonable form, then they have discharged their obligations and the consent is informed (in that the information has been provided) even though understanding is not guaranteed. If we took this route then there would not be an ethical consent problem, but there are at least two reasons for not having such a minimalist view of consent. First, that is not how valid consent is generally understood these days. And second, it is not clear what the point of providing the information is, or indeed what the point of informed consent is, if we take little or no interest in whether the recipients of that information have understood it.

A different approach to a parallel issue is explored in a paper by Dawson on informed consent in clinical trials. Dawson enquired about the implications, for the ethics of consent, of the fact that many trial participants seem not to be able to grasp important aspects of the research process, in particular randomization. Dawson offers what he calls the Unattainability Argument, which goes as follows.

1. It is claimed that we ought to obtain IC [informed consent] from competent adult research participants
2. We cannot obtain IC in a significant number of cases
3. Where we cannot actually obtain IC, it makes no sense to require it.
  Conclusion: It makes no sense to require IC.^32^

It does not take much imagination to see how one might construct a parallel argument for PNS –

- It is claimed that we ought to obtain informed consent from pregnant women before undertaking PNS
- We cannot obtain informed consent in a significant number of cases
- Where we cannot actually obtain informed consent, it makes no sense to require it.
  Conclusion: It makes no sense to require informed consent for (some/many types of) PNS.

This, then, is one way of responding to the impossibility of understanding. If understanding is impossible, and if understanding is required for informed consent, then informed consent cannot be ethically required. For ‘ought implies can’: we cannot be morally required to do something impossible.

So what impact do these concerns about information and understanding have on the ethics of PNS?

The ‘first line’ response to such consent worries must always be to work hard on making clinical practice as good as it can be, in terms of making the information as accessible and digestible as possible for pregnant women who are offered tests. But let's allow, for the sake of argument at least, that this sometimes fails. What then should we say?

The first thing to keep in mind is that these concerns, especially those relating to the comprehension of probability and risk, are not unique to PNS. Dawson, as mentioned earlier, argues that similar issues arise in biomedical research and I would add that *if* such claims about people's inability to understand are true, they will affect a very wide range of everyday life choices, including for example people's capacity to make adequately informed choices about important financial services (insurance, loans, mortgages, etc.). While we have reason to be concerned about the quality of choice and consent in PNS, as in other areas of healthcare, it looks as if the position of PNS is not obviously worse than that of other important areas of life.

Which of the two models can cope best with this consent issue: Pure Choice or Public Health Pluralism? Pure Choice will be especially vulnerable to consent problems for, on this view, the sole reason for providing PNS is to give choice. But if these choices (and consents) are ‘defective’ (because based on information which is not understood) and hence not meaningful or properly autonomous, the whole point of providing screening seems to be undermined. Public Health Pluralism, on the other hand, may be better placed to withstand consent problems, since – while it values choice and consent – it appeals to other goals and values too.

So, to conclude, it can be argued that if governments or national health services seek to reduce the prevalence of disability and disease in future generations by encouraging PNS, this will create additional cases in which pregnant women's consents (to screening and testing, and/or to termination) are defective or invalid.

Against this, I argued initially that the consent problem is not as bad, or at least not as intractable, as is sometimes supposed. One reason for this is a certain degree of optimism about our ability to get pregnant women to understand the tests on offer. A second is the suggestion that (if the concern is about people's ability to understand probability) PNS is in no worse a position than (say) financial services or gambling. Yet another reason is the argument (inspired by Dawson) which says that, if our consent requirements cannot possibly be met, then we would be well-advised either to modify the information standards (so that they *can* be met) or, more radically, to drop the informed consent requirement altogether, rather than concluding that PNS is all irredeemably unethical.

Furthermore, even if it is conceded that there is a consent issue, that pregnant women are often unable to give informed consent to PNS, this does not count against Public Health Pluralism. For consent would then be a problem area for PNS regardless of which model was adopted. Furthermore, it is liable to be a bigger problem for Pure Choice than for Public Health Pluralism since – whereas Public Health Pluralism values autonomy and choice amongst several other things – for Pure Choice, the fundamental reason for providing PNS is to enable choice. So if the choices made and consents provided turn out to be somehow ‘defective’ and hence not meaningful or properly autonomous, the whole point of providing screening is undermined. Public Health Pluralism, on the other hand, may still be able to defend at least some kinds of PNS on grounds other than choice.

So either the consent issue is surmountable, in which case there is no (major) consent problem for either side in the debate, or it is insurmountable, in which case it is a problem for both Public Health Pluralism *and* Pure Choice, but arguably more so for the latter. Interestingly this general conclusion also applies to other consent arguments not considered in detail here: such as the claim that pregnant women's consents to screening and termination are vitiated by general social pressure to avoid having a child with a disability (in the form of, for example, lack of financial support for families with disabled children, or lack of access to health and social services).

## 5. Conclusion

This article critiques the Pure Choice rationale for state-supported PNS by comparing it with a possible alternative, Public Health Pluralism. It argues that we have reasons to prefer the latter including the following. First, Public Health Pluralism does not (as is often supposed) render PNS more vulnerable to objections couched in terms of eugenics. Second, the Pure Choice view, if followed through to its logical consequences, may have some unpalatable implications, such as extending choice well beyond health screening. Third, any sensible version of Public Health Pluralism (and any ethical PNS programme) will take on board the moral seriousness and sensitivity of abortion and will advocate, where practicable, alternative means of reducing the prevalence of disease and disability; but where there is no practicable alternative, facilitating selective abortion may still be justified. Hence, Public Health Pluralism is not especially vulnerable to criticisms based on the undesirability of abortion. Finally, Public Health Pluralism is entirely consistent with valuing autonomy and consent and is not especially vulnerable to consent problems. Indeed, as we have just seen, if consent is a ‘problem area’ for PNS then this will be a problem for both Public Health Pluralism *and* Pure Choice, but arguably it is an even bigger problem for the latter.
